# Chemical Compounds of Malacca Leaf (*Phyllanthus emblica*) after Triple Extraction with N-Hexane, Ethyl Acetate, and Ethanol

**DOI:** 10.1155/2020/2739056

**Published:** 2020-04-27

**Authors:** Nuzul Asmilia, Yudha Fahrimal, Mahdi Abrar, Rinidar Rinidar

**Affiliations:** ^1^Graduate School of Mathematics and Applied Sciences, Universitas Syiah Kuala, Banda Aceh 23111, Indonesia; ^2^Clinical Laboratory, Faculty of Veterinary Medicine, Universitas Syiah Kuala, Banda Aceh 23111, Indonesia; ^3^Parasitology Laboratory, Faculty of Veterinary Medicine, Universitas Syiah Kuala, Banda Aceh 23111, Indonesia; ^4^Microbiology Laboratory, Faculty of Veterinary Medicine, Universitas Syiah Kuala, Banda Aceh 23111, Indonesia; ^5^Pharmacology Laboratory, Faculty of Veterinary Medicine, Universitas Syiah Kuala, Banda Aceh 23111, Indonesia

## Abstract

Malacca (*Phyllanthus emblica*) is one of the plants that is often by the community in the Aceh Besar district of Indonesia as a traditional medicine for the treatment of various diseases such as antimicrobial, antibacterial, antifungals, antivirals, antimutagenic, antimalaria, and antiallergic. This research was conducted to analyze the content of chemical compounds in the ethanol extract of the Malacca leaf (EEDM) using a gas chromatography-mass spectrophotometer (GC-MS). Malacca leaves were extracted by the maceration method using n-hexane, ethyl acetate, and ethanol. The GC-MS analysis showed EEDM contained 22 chemical compounds. The highest chemical content of EEDM is octadecanoic acid reaching 22.93%, 9,12-octadecanoic acid 14.99%, octadecanoic acid 7.59%, 9-hexadecenoic acid 6.17%, octadecanoic acid 5.95%, octadecanal 5.59%, 9,12-octadecanoic acid 5.06%, 3-eicosyne 4.75%, 1-hexadecenoic acid 4.08%, 11-tetradecen-1-ol 2.92%, 2-furanmethanol 2.83%, delta-guaiene 2.43%, cyclohexane 2.13%, hexadecanoic acid 1.99%, sativen 1.87%, octadecanoic acid 1.52%, 1H-cyclopropaanaphthalene 1.40%, tetradecanoic acid 1.40%, 3,7,11-tridecatrienenitrile 1.20%, caryophellene 1.11%, 2H-pyran 1.07%, and trans-caryophellene 1.03%. This study clearly shows the presence of fatty acids which play a major role in the efficacy of these traditional medicines particularly as antioxidant and antimalarial.

## 1. Introduction

Malacca (*Phyllanthus emblica*) is one of the plants that is often used by the community in the Aceh Besar district of Indonesia as a traditional medicine for the treatment of various diseases. *Phyllanthus emblica* in Indonesia is known as the kimalaka [1]. The plant is also called “bak rem“ (Acehnese), balakka in North Sumatra, metengo in Ternate [2], and kemloko in Java [3]. In English, these plants are referred as gooseberry Indians [4], and India is known by various names such as aonla, nelli, amla, amlika, dhotri, emblica, and usuri [5] In Malaysia, it is called diaper melaka, in German called amla [6], and in Thailand called ma-kham-pom [7].

Malacca is widespread in most tropical countries and subtropical countries and is native to South and Southeast Asia [8]. This plant belongs to the family Euphorbiaceae which is widely distributed and grows wild in India, Sri Lanka, Pakistan, Uzbekistan, China, Indonesia, Malaysia, and Thailand. Malacca can grow on the hillside with a height of 200 meters and can be cultivated [9, 10]. The identification of *Phyllanthus emblica* Linn., has a very large genus of around 550 to 750 species which are divided into 10 to 11 subgenera [11].

Numerous studies have been done on the Malacca plant which reported that the Malacca plant has a potency as chemoprotective and antioxidant [6], analgesic, antipyretic [12], antitumor [13, 14], anti-inflammation [15, 16], able to decrease blood sugar level [17], antiviral, antimutagenic, and antiallergic [18], antimicrobial, and antibacterial antifungals [19, 20]. Malar and Mary [21] proved that the fruit of Malacca can appear to be able to repair liver damage, and Asmilia et al. [22] reported that the ethanolic extract of Malacca leaves has a higher inhibition level on *Plasmodium falciparum* growth compared to ethyl acetate and n-hexane extract of Malacca leaves. Deepak and Gopal [23] found terpenes, phytosterols, and terpenoids in ethyl acetate extracts from the bark of *Phyllanthus emblica.* So far, there has been no report on the content of the chemical compounds of Malacca leaves triple extracted using n-hexane, ethyl acetate, and ethanol solution. Therefore, analysis and determination of the content of the chemical compounds of Malacca leaves need to be done. This study was conducted to determine the content and identification of the ethanol extract compound of Malacca leaves macerated in three stages using n-hexane, ethyl acetate, and ethanol.

## 2. Materials and Methods

### 2.1. Malacca Leaf Samples

Malacca leaf samples were collected from Malacca plants that grew in the Blang Thutu area, Lambirah Village, Aceh Besar, Aceh, in April 2018. Identification of plants was conducted at the Herbarium Biology of Faculty of Mathematics and Natural Sciences, Syiah Kuala University, and identified as the *Phyllanthus emblica* Linn.

### 2.2. Malacca Leaf Sample Preparation

The extraction was carried out at Pharmacology Laboratory, Faculty of Veterinary Medicine, Syiah Kuala University. Malacca leaf samples were dried for several days, and then 1.5 kg dried samples were mashed to form simplisia. Simplisia was put into Erlenmeyer and then added with 1 liter of n-hexane. The maceration of simplisia was carried out for three days, and stirring was done infrequently. Subsequently, the maceration solution was filtered using the filter paper, and then the precipitate of simplisia was added with ethyl acetate followed by the maceration process for three days and filtration of the residue. The third maceration was performed using ethanol for three days, filtered, and dried using an evaporator until reaching paste-like texture.

### 2.3. Identification of Chemical Compounds by Using Gas Chromatography-Mass Spectrophotometer

Gas chromatography-mass spectrophotometer (GC-MS) used was GC-MS-QP2010S SHIMADZU consisting of the AOC-20i autosampler and gas chromatography connected to mass spectrometer instruments (GC-MS) using the following conditions: Restek RtxR-5 (30 meters × 0.25 mm) (5% diphenyl and 95% dimethyl polysiloxane) runs in the electron impact mode at 70 eV; helium (99.99%) was used as a carrier gas at a constant flow of 1 ml/minute, and an injection volume of 1.0 *μ*l was used (split ratio 10 : 1); and 300°C injector temperature. The oven temperature was programmed from 70°C (isothermal for 5 minutes), with an increase of 6°C/minute to 280°C and then terminated with isothermal for 15 minutes at 280°C. The mass spectrum was taken at 70 eV; 0.5 second scanning interval; and fragments from 40 to 550 Da. The total running time of GC is 60 minutes.

The peaks that appear on the GC-MS screen were interpreted using the National Institute of Standard and Technology (NIST) database. The mass spectrum of unknown components was compared to the spectrum of known components stored in the NIST library.

## 3. Results and Discussion

**Figure 1:**
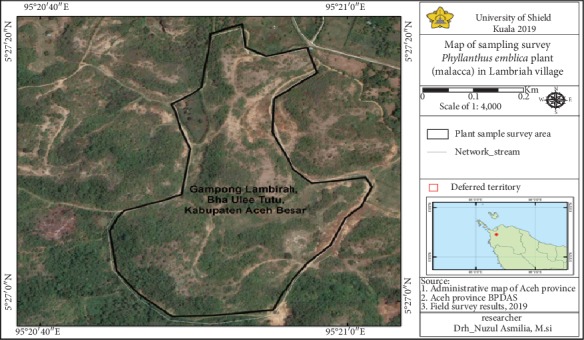
Sampling location.

Malacca leaf samples were collected from the Malacca plant which grew in Blang Thutu, Lambirah Village of Aceh Besar district (Figure 1).

**Figure 2:**
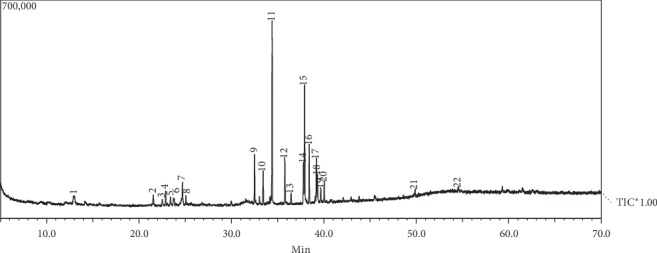
GC-MS chromatogram of the ethanolic extract of the leaves of *Phyllanthus emblica* leaves.

The chemical compounds of the ethanolic extract of the Malacca leaf identified using GC-MS can be seen in Figure 2. Structural analysis of organic compounds spectroscopically included using a mass spectrum to determine the molecular formula and to determine the mass of a molecule [24]; in addition to GCMS, there is also an infrared spectrophotometer to determine the functional group of a compound [25]. There were 22 chemical compounds identified, and the most abundant compound in the Malacca ethanolic extract of Malacca leaves was decanoic acid (22.93%).

Octadecanoic acid was detected at 34.4 minutes in the ethanol extract of Malacca leaves and has another name stearic acid which has 18 carbon atom chain and saturated fatty acid that has a carbon backbone and consists of a long chain of carbon atoms bonded together by single bonds and with 2 hydrogen atoms attached to each internal carbon atom. The second largest compound detected at minute 37.9 was 9,12-octadecadinoic acid (14.99%). The third most detected compound at minute 38.4 was octadecanoic acid (7.59%), and the next minute was 9-hexadecenoic acid (6.17%), octadecanoic acid (5.95%), octadecanal (5.59%), 9,12-octadecanoic acid (5.06%), 3-eicosyne (4.75%), 1-hexadecenoic acid (4.08%), 11-tetradecen-1-ol (2.92%), 2-furanmethanol (2.83%), delta-guaiene (2.43%), cyclohexane (2.13%), hexadecenoic acid (1.99%), sativen (1.87%), octadecanoic acid (1.52%), 1H-cyclopropaanaphthalene (1.40%), tetradecanoic acid (1.40%), 3,7,11-tridecatrienenitrile (1.20%), caryophellene (1.11%), 2H-pyran (1.07%), and minute 23.4 found trans-caryophellene (1.03%) (Table 1).

Some types of medicinal plants that contain a lot of fatty acids are very good as antioxidants and antimalarial. The active ingredients detected have very little detailed information available explaining the function of each of them. Another name for tetradecanoic acid is myristic acid, the dodecanoic acid synonym is lauric acid, and the synonym of octadecanoic acid is stearic acid; the whole chemical component is included in the group of saturated fatty acids [26].

## 4. Conclusions

The GC-MS analysis of the Malacca leaf extract (*Phyllanthus emblica*) clearly shows the presence of fatty acids which are mainly found in the leaf. There were 22 chemical compounds identified, and the most abundant compound in the Malacca ethanolic extract of Malacca leaves was decanoic acid (22.93%). These chemical constituents play a major role in the efficacy of these traditional medicines particularly as antioxidants and antimalarial. The study also showed that many other phytochemical compounds have not been tested for their biological activity. Therefore, further research is needed to be carried out on the application of these phytochemical compounds in pharmaceutical fields.

## Figures and Tables

**Table 1:** Phytocomponents identified from the ethanol extract of *Phyllanthus emblica* leaves.

Peak no.	Retention time	Name of the compound	Molecular formula	Mol weight	Peak area %
1	12,949	2-Furanmethanol	C5 H6 O2	98	2.83
2	21,551	1H-Cyclopropaanaphthalene	C15 H24	204	1.40
3	22,514	Trans-caryophyllene	C15H24	204	1.03
4	22,874	Cyclohexane	C13H20	176	2.13
5	23,402	Caryophyllene	C15H24	204	1.11
6	23,402	Sativen	C15H24	204	1.87
7	23,794	Delta-guaiene	C15H24	204	2.43
8	24,695	Tetradecanoic acid	C15H30O2	242	1.40
9	32,500	Octadecanal	C18H36O	268	5.59
10	33,428	3-Eicosyne	C20H38	278	4.75
11	34,402	Octadecanoic acid	C19H38 O2	298	22.93
12	35,784	Octadecanoic acid	C20H40 O2	312	5.95
13	36,444	Octadecanoic acid	C19H38 O2	298	1.52
14	37,792	9,12-Octadecadienoic acid	C19H34 O2	294	5.06
15	37,921	9,12-Octadecadienoic acid	C19H34 O2	294	14.99
16	38,414	Octadecanoic acid	C19H38 O2	298	7.59
17	39,184	9-Hexadecenoic acid	C16H30 O2	254	6.17
18	39,304	1-Hexadecanol	C16H34O	242	4.08
19	39,668	Hexadecenoic acid	C18H36 O2	284	1.99
20	40,034	11-Tetradecen-1-ol	C16H30 O2	254	2.92
21	49,859	3,7,11-Tridecatrienenitrile	C16H25N	231	1.20
22	54,548	2H-Pyran	C22H40 O2	336	1.07

## Data Availability

The data used to support the findings of this study are available from the corresponding author upon request.
